# Dried and Fermented Powders of Edible Algae (*Neopyropia yezoensis*) Attenuate Hepatic Steatosis in Obese Mice

**DOI:** 10.3390/molecules27092640

**Published:** 2022-04-20

**Authors:** Koji Nagao, Nao Inoue, Keisuke Tsuge, Akira Oikawa, Tomoko Kayashima, Teruyoshi Yanagita

**Affiliations:** 1 Department of Biological Resource Science, Saga University, 1 Honjo-machi, Saga 840-8502, Japan; d5589@cc.saga-u.ac.jp (N.I.); yanagitt@cc.saga-u.ac.jp (T.Y.); 2 The United Graduate School of Agricultural Sciences, Kagoshima University, Kagoshima 890-0065, Japan; 3 Saga Regional Industry Support Center, Saga 849-0932, Japan; tsuge@saga-itc.jp; 4 Graduate School of Agriculture, Kyoto University, Uji 611-0011, Japan; oikawa.akira.7j@kyoto-u.ac.jp; 5 Faculty of Education, Saga University, 1 Honjo-machi, Saga 840-8502, Japan; kaya@cc.saga-u.ac.jp

**Keywords:** *Neopyropia yezoensis*, Koji fermentation, hepatic steatosis, obese *db*/*db* mouse, eicosapentaenoic acid, stachydrine, betaine, carnitine

## Abstract

Edible algae *Neopyropia yezoensis* is used as “Nori”, its dried sheet product, in Japanese cuisine. Its lipid components reportedly improve hepatic steatosis in obese *db*/*db* mice. In this study, we prepared “Nori powder (NP)” and “fermented Nori powder (FNP)” to utilize the functional lipids contained in “Nori” and examined their nutraceutical effects in vivo. Male *db*/*db* mice were fed a basal AIN-76 diet, a 10% NP-supplemented diet, or a 10% FNP-supplemented diet for 4 weeks. We detected eicosapentaenoic acid (EPA) present in both NP and FNP in the serum and liver of *db*/*db* mice in a dose-dependent manner. The NP diet reduced hepatic triglyceride accumulation (by 58%) in *db*/*db* mice by modulating gene expression, which resulted in the inhibition of lipogenic enzyme activity. Additionally, NP intake significantly suppressed the expression of inflammatory genes in the liver and hepatic injury marker levels in the sera (by 26%) of *db*/*db* mice. The FNP diet also led to a marked reduction in hepatic triglyceride accumulation (by 50%) and hepatic injury (by 28%) in *db*/*db* mice, and the mechanism of these alleviative actions was similar to that of the NP diet. Although the EPA content of FNP was one-third that of NP, metabolomic analysis revealed that bioactive betaine analogs, such as stachydrine, betaine, and carnitine, were detected only in FNP. In conclusion, we suggest that (1) mechanical processing of “Nori” makes its lipid components readily absorbable by the body to exert their lipid-lowering effects, and (2) fermentation of “Nori” produces anti-inflammatory molecules and lipid-lowering molecules, which together with the lipid components, can exert hepatic steatosis-alleviating effects.

## 1. Introduction

Obesity is the central pathology of the metabolic syndrome, a cluster of metabolic abnormalities that contributes to increased cardiovascular morbidity and mortality in developed countries [1,2]. Non-alcoholic fatty liver disease (NAFLD) is a spectrum of conditions ranging from hepatic steatosis to steatohepatitis, advanced fibrosis, and cirrhosis. The presence of metabolic syndrome is associated with potentially progressive severe liver disease [3,4]. Liver-related morbidity and mortality due to NAFLD are mostly observed in patients with advanced fibrosis and cirrhosis. Therefore, the effective use of dietary factors to alleviate hepatic steatosis, the early NAFLD pathogenesis, and preventing its worsening can solve a serious public health problem. *db*/*db* mice, which have leptin receptor gene mutations, share many features with human metabolic syndrome [5,6,7]. These mice are well-suited for evaluating how dietary components affect the development of obesity-induced hepatic steatosis [8].

The nutritional value and bioactive components of algae have been reported to be useful for improving health, and therefore the laver industry is expected to develop it as a source of edible seaweed and useful compounds [9]. The edible seaweed *Neopyropia yezoensis*, formerly known as *Porphyra yezoensis* or *Pyropia yezoensis*, is dried into sheets as “Nori” and used to wrap sushi and rice balls in Japanese cuisine. These edible red algae, recognized as one of the most delicious seaweeds and are stably obtained, are used not only in Japan but also worldwide. Nori is rich in nutrients and its lipid components include eicosapentaenoic acid (EPA) bound to glycolipids and phospholipids [10,11,12]. We previously reported that complex lipids extracted from Nori improved hepatic steatosis in obese *db*/*db* mice [12]. However, since the membrane lipid components of seaweed are surrounded by the cell wall, it is assumed that humans cannot utilize the lipid components of Nori if they eat it as is.

Therefore, we prepared “Nori powder (NP)” by mechanical processing and aimed to make the functional lipids in Nori bioavailable by cell wall fragmentation. Additionally, since a previous report indicated that Koji fermentation of Nori releases EPA and the degrades cell wall polysaccharides [13], we also prepared “fermented Nori powder (FNP)” as a biochemically processed product. In this study, incorporation of EPA from these dried powders, NP and FNP, and their nutraceutical effects were evaluated in vivo using obese *db*/*db* mice.

## 2. Results

### 2.1. Preparation and Characterization of Nori Powder and Fermented Nori Powder

**Figure 1:**
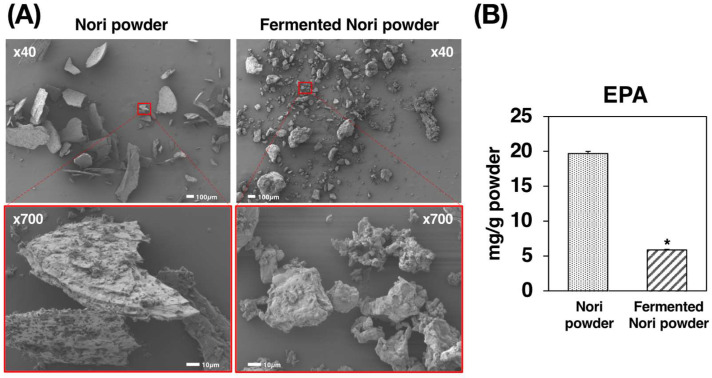
(**A**) Scanning electron micrographs and EPA contents of NP and FNP. (**B**) Values are expressed as the mean ± standard error (*n* = 3). Asterisk shows significant difference (*p* < 0.05) between the two powders. NP: Nori powder, FNP: fermented Nori powder, EPA: eicosapentaenoic acid.

We used scanning electron microscopy (SEM) to confirm the physical and biochemical breakdown of Nori (Figure 1A). The NP prepared by mechanical processing retained its cell wall structure, suggesting that digestive enzymes can act on the cross-section in vivo. However, the prepared FNP, biochemically processed by Koji fermentation, had a less distinct cell wall structure and a smaller size due to the *Aspergillus oryzae* growth and digestion. Interestingly, upon measuring the EPA content by GC-MS, we found that it was 19.7 mg/g and 5.87 mg/g in the NP and FNP, respectively (Figure 1B, Supplementary Table S1).

### 2.2. Effects of Experimental Diets on Growth Parameters

To assess the impact of the sample powder ingestion, we set up three test groups. We divided the *db*/*db* mice into three groups that were fed one of three diets: (1) a basal semisynthetic AIN-76 diet (Co group, *n* = 5); (2) a semisynthetic AIN-76 diet supplemented with 10% NP at the expense of sucrose (Np group, *n* = 6); or (3) a semisynthetic AIN-76 diet supplemented with 10% FNP at the expense of sucrose (Fp group, *n* = 6). After 4 weeks of feeding the mice with the experimental diets, we found that the three groups did not differ in initial body weight, final body weight, weight gain, food intake, and abdominal white adipose tissue (WAT) weights. No differences in feed preference or side effects, such as diarrhea, were observed. However, the Np and the Fp groups displayed significantly reduced liver weights as compared with the Co group, as shown in Table 1.

### 2.3. Incorporation of EPA in the Serum and Liver of db/db Mice

**Figure 2:**
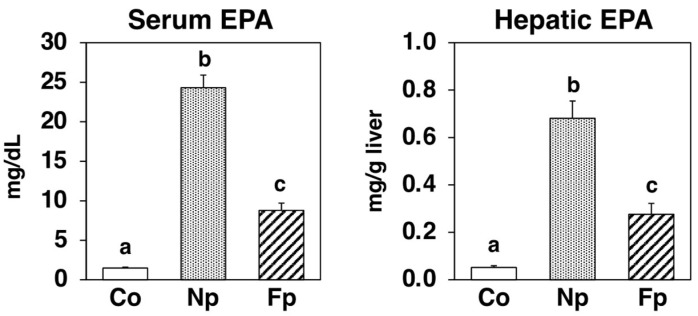
EPA levels in the sera and livers of *db*/*db* mice fed a basal diet (Co group), 10% NP-supplemented diet (Np group), or 10% FNP-supplemented diet (Fp group) for 4 weeks. Values are expressed as the mean ± standard error (*n* = 5–6). ^abc^ Different letters denote significant differences (*p* < 0.05) among the three groups. EPA: eicosapentaenoic acid.

After a 4-week feeding period, the EPA in the serum and liver was 1.48 mg/dL and 0.0515 mg/g liver, respectively, in the Co group (without dietary EPA). However, in the Np group, EPA in the serum and liver was 24.3 mg/dL and 0.681 mg/g liver, respectively, while in the Fp group, it was 8.78 mg/dL and 0.276 mg/g liver, respectively. These results indicate that both physical and biochemical breakdown of Nori makes its lipid components readily absorbable by the body, with the EPA being detected in each mice group reflecting the relative EPA content of the diets (Figure 2, Supplementary Tables S1–S3).

### 2.4. Effects of Experimental Diets on Liver Histology and Triglyceride Levels

**Figure 3:**
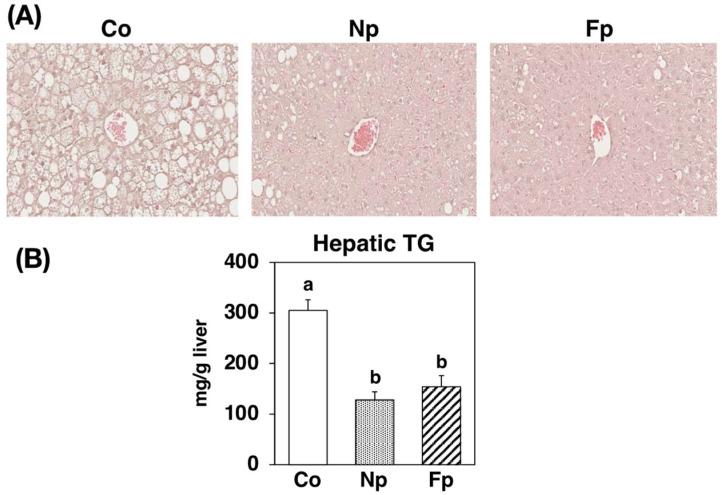
Liver histology and triglyceride levels in *db*/*db* mice fed a basal diet (Co group), 10% NP-supplemented diet (Np group), or 10% FNP-supplemented diet (Fp group) for 4 weeks. (**A**) Hematoxylin and eosin staining of liver sections from mice representative of each experimental group. (**B**) Values are expressed as the mean ± standard error (*n* = 5–6). ^ab^ Different letters denote significant differences (*p* < 0.05) among the 3 groups. TG: triglyceride.

We observed macrovesicular hepatocytes and hepatic triglyceride accumulation in obese *db*/*db* mice in the Co group (Figure 3). However, these were markedly alleviated in both the Np and Fp groups as compared with the Co group.

### 2.5. Effects of Experimental Diets on Hepatic Expression of Genes Related to Lipid Metabolism

**Figure 4:**
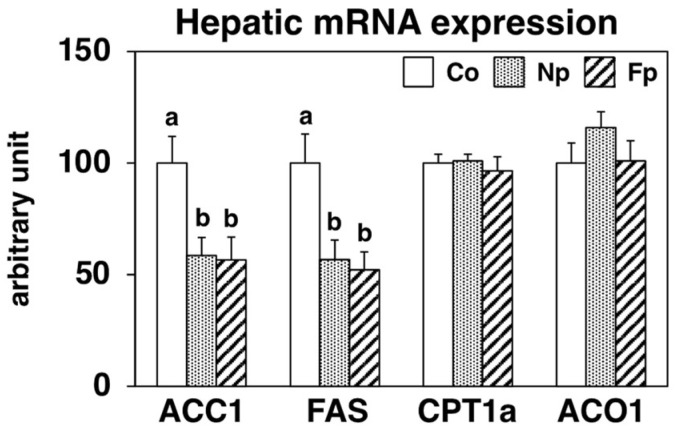
mRNA expression levels of genes related to lipid metabolism in the liver of *db*/*db* mice fed a basal diet (Co group), 10% NP-supplemented diet (Np group), or 10% FNP-supplemented diet (Fp group) for 4 weeks. Values are expressed as the mean ± standard error (*n* = 5–6). ^ab^ Different letters denote significant differences (*p* < 0.05) among the three groups. ACC1: acetyl-CoA carboxylase-1, FAS: fatty acid synthase, CPT1a: carnitine palmitoyltransferase-1a, ACO1: acyl-CoA oxidase-1.

To elucidate the hepatic steatosis alleviating effects of the experimental diets, we analyzed the hepatic expression of genes related to lipid metabolism using real-time PCR (Figure 4). Concomitant with decreased triglyceride levels, the hepatic mRNA expression of lipogenic enzymes like acetyl-CoA carboxylase-1 (ACC1) and fatty acid synthase (FAS) was significantly suppressed in both the Np and Fp groups as compared with that of the Co group. In contrast, among groups, there was no significant difference in the mRNA levels of carnitine palmitoyltransferase-1a (CPT1a), a key enzyme of mitochondrial fatty acid β-oxidation, and acyl-CoA oxidase-1 (ACO1), a key enzyme of peroxisomal fatty acid β-oxidation.

### 2.6. Effects of Experimental Diets on Activities of Enzymes Related to Lipid Metabolism in the Liver

**Figure 5:**
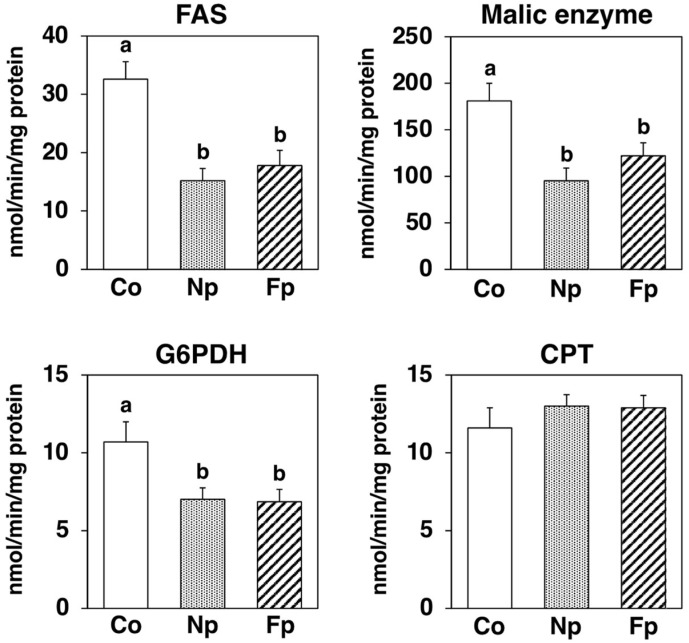
Activities of hepatic enzymes related to lipid metabolism in *db*/*db* mice fed a basal diet (Co group), 10% NP-supplemented diet (Np group), or 10% FNP-supplemented diet (Fp group) for 4 weeks. Values are expressed as the mean ± standard error (*n* = 5–6). ^ab^ Different letters denote significant differences (*p* < 0.05) among the three groups. FAS: fatty acid synthase, G6PDH: glucose-6-phosphate dehydrogenase, CPT: carnitine palmitoyltransferase.

To further examine the effects of the powders on the liver, we measured the activities of key enzymes related to lipid metabolism (Figure 5). In the Np and the Fp groups, the activities of FAS (a key enzyme for the de novo synthesis of fatty acids) significantly decreased as compared with that of the Co group. Additionally, the activities of both malic enzyme and glucose-6-phosphate dehydrogenase (G6PDH), which supplies nicotinamide adenine dinucleotide phosphate required for FAS activity, significantly decreased in the Np and the Fp groups as compared with the Co group. There was no significant difference in the activity of CPT, a key enzyme in mitochondrial fatty acid β-oxidation, among groups.

### 2.7. Effects of Experimental Diets on Hepatic Inflammatory and Injury Marker Levels

**Figure 6:**
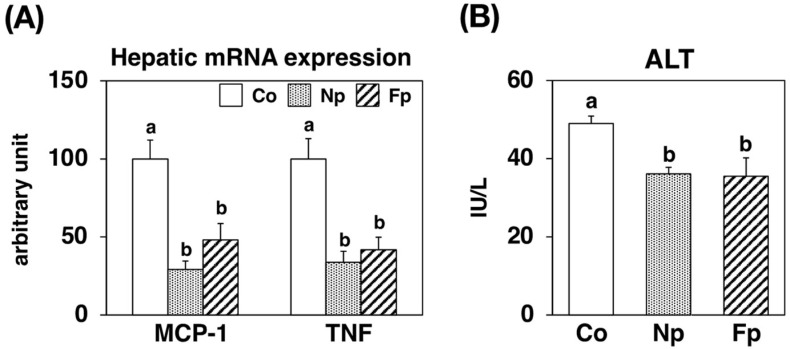
mRNA expression levels of genes related to inflammation in the liver and the hepatic injury marker levels in *db*/*db* mice fed a basal diet (Co group), 10% NP-supplemented diet (Np group), or 10% FNP-supplemented diet (Fp group) for 4 weeks. Values are expressed as the mean ± standard error (*n* = 5–6). ^ab^ Different letters denote significant differences (*p* < 0.05) among the three groups. MCP-1: monocyte chemoattractant protein-1, TNF: tumor necrosis factor, ALT: alanine aminotransferase.

To gain an insight into the effect of the powders on the liver, we also examined the hepatic mRNA expression of genes related to inflammatory and hepatic injury marker levels in the serum (Figure 6). Compared with the Co group, the mRNA expression of monocyte chemoattractant protein-1 (MCP-1) and tumor necrosis factor α (TNFα), which exacerbate chronic inflammation and injury in the liver, was markedly decreased in both the Np and Fp groups. Moreover, the activity of the hepatic injury marker alanine aminotransferase (ALT) in the serum was significantly lower in both the Np and Fp groups than in the Co group.

### 2.8. Contribution of EPA Incorporation into the Liver on the Alleviation of Hepatic Injury

**Figure 7:**
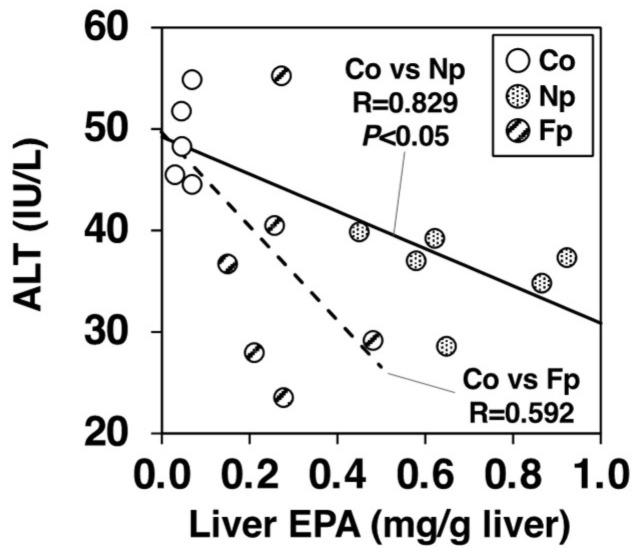
Correlations between hepatic injury marker levels (ALT activities) in the sera vs EPA levels in the livers of *db*/*db* mice. The mice were fed a basal diet (Co group), 10% NP-supplemented diet (Np group), or 10% FNP-supplemented diet (Fp group) for 4 weeks. Individual values are shown (*n* = 17). EPA: eicosapentaenoic acid, ALT: alanine aminotransferase.

To further investigate the ameliorating effects of each powder on liver damage, we analyzed how hepatic EPA incorporation reduced hepatic injury markers using a single regression analysis between the Co and Np groups and between the Co and Fp groups (Figure 7). Liver EPA levels exhibited a significant negative correlation with hepatic injury markers in the sera of *db*/*db* mice (ALT vs. EPA, r = 0.829, *p* < 0.05) between the Co and Np groups. However, there was no statistically significant correlation between hepatic EPA incorporation and ALT reduction (r = 0.592) between the Co and Fp groups. These results suggest that there are additional factors to EPA incorporation, by which the FNP diet effectively attenuates hepatic injury in *db*/*db* mice.

### 2.9. Metabolomic Changes in Nori Powder before and after Koji Fermentation

**Figure 8:**
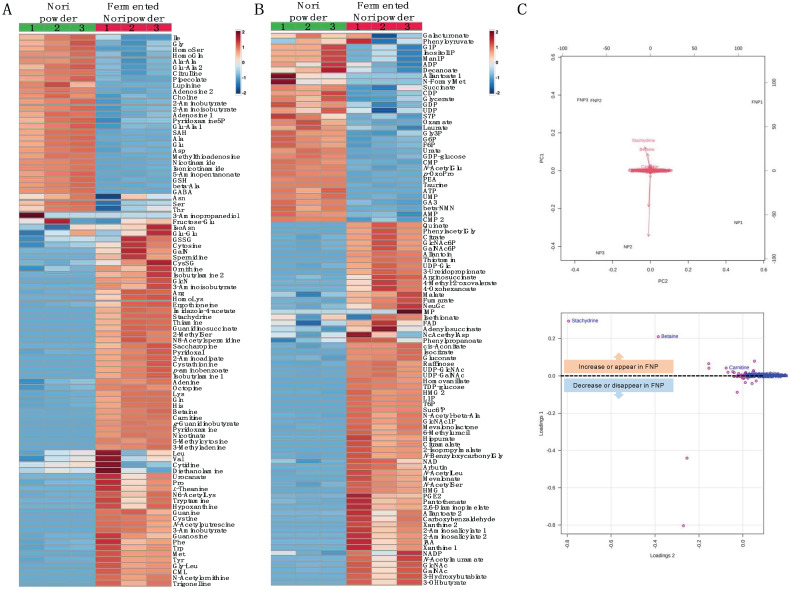
Heat map analysis and principal component analysis of the 189 metabolites significantly modified by Koji fermentation. (**A**: Cationic mode, **B**: Anionic mode) The normalized values were transformed into a z-score and shown as a color scale. The high and low metabolite levels were represented as reddish and blueish scales, respectively. (**C**) Principal components analysis: biplot (upper figure) and loading plot (lower figure) from metabolites of powders before and after Koji fermentation. Labeled variables were detected only in FNP. NP: Nori powder, FNP: fermented Nori powder.

**Figure 9:**
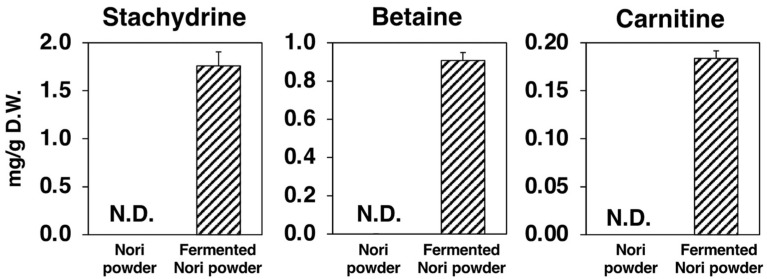
Amounts of representative metabolites of each group. Values are expressed as the mean ± standard error (*n* = 3). N.D.: not detected.

We performed metabolomic analyses on NP and the FNP to examine differences in metabolites before and after Koji fermentation. We identified and quantified 93 metabolites in the cationic mode of CE-TOF MS analysis, of which 32 and 61 were decreased and increased, respectively, by Koji fermentation (Figure 8A). Ninety-six metabolites were identified and quantified in the anionic mode of CE-TOF MS analysis, of which 33 and 63 were decreased and increased, respectively, by Koji fermentation (Figure 8B). Of the 189 metabolites, we lost 4 and newly detected 64 after Koji fermentation. As shown in the biplots and loading plots of the principal component analysis (PCA), Koji fermentation promoted the synthesis of three betaine structural analogues: stachydrine, betaine, and carnitine (Figure 8C, labeled variables were detected only in FNP). The results showed that 1.76 mg/g (9.80 μmol/g), 0.908 mg/g (7.75 μmol/g) and 0.184 mg/g (1.14 μmol/g) of stachydrine, betaine and carnitine, respectively, were markedly synthesized through Koji fermentation (Figure 9).

## 3. Discussion

EPA (C20:5, ω3) is one of the most reliable functional fatty acids which reportedly prevent cardiovascular diseases [14]. Its ethyl ester agents (Epadel, Vasepia) are used as treatments for hyperlipidemia [14,15]. The dried sheet product of *Neopyropia yezoensis,* Nori, has a characteristic fatty acid composition, consisting mostly of EPA (>50% of total fatty acids) and palmitic acid (C16:0, ~30% of total fatty acids). However, the EPA in Nori is not a component of storage lipids like free fatty acids or triglycerides but of the cell membrane structural lipids like glycolipids and phospholipids [10,11,12]. Although it has been reported that the intestinal bacteria of some Japanese people have enzymes to digest seaweed [16], we hypothesized that the Nori in powder form could increase the bioavailability of EPA in Nori by cell wall fragmentation (Figure 1A). After 4 weeks of feeding the NP-supplemented diet, significant amounts of EPA were detected in the sera and livers of *db*/*db* mice, suggesting that the mechanical processing of Nori promotes the in vivo uptake of its lipid components (Figure 2). In addition, we also performed Koji fermentation for the biochemical processing of Nori. Koji fermentation using *Aspergillus oryzae* is essential for the production of many traditional Japanese foods, like miso, soy sauce, and sake [17,18,19]. Consistent with previous studies indicating that Koji fermentation of Nori degraded cell wall polysaccharides and liberated EPA [13], the structure of Nori was indeed found broken down by fermentation (Figure 1A), and more EPA was detected in the sera and livers of the Fp group than in the Co group (Figure 2). However, the reason that the EPA content per dry weight of FNP was one-third that of NP is not yet known (Figure 1B). However, it was also shown that the EPA content of the powders was reflected in the amounts of EPA incorporated into the body (Figure 2).

In this study, in addition to confirming the uptake of EPA from the prepared powders, we also studied the effects of ingesting these powders on NAFLD development in obese *db*/*db* mice. After 4 weeks of feeding, the Np group showed no change in WAT weight, but the liver weight and liver triglyceride levels were significantly reduced compared with those in the Co group (Table 1, Figure 3). To investigate the mechanism of the hepatic lipid-lowering effect, we first examined its effect on the expression of genes related to lipid metabolism in the liver, and we found the NP diet significantly decreased the mRNA levels of ACC1 and FAS (Figure 4). The transcription factor sterol regulatory element binding protein (SREBP)-1 regulates the gene expression of these lipogenic enzymes, and interestingly ω3 polyunsaturated fatty acids (PUFAs) exert their lipid-lowering effects by inhibitory SREBP-1 signaling [20]. Furthermore, consistent with the inhibitory effect on lipid synthesis at the gene expression level, the activities of lipogenic enzymes (FAS, G6PDH and Malic Enzyme) in the liver were significantly reduced in the Np group as compared with the Co group (Figure 5). However, whether EPA was the sole lipid synthesis inhibitory component incorporated from NP in this experiment needs further exploration. Although EPA has been reported to act as a ligand for peroxisome proliferator-activated receptor-α, a transcription factor that regulates the expression of lipolytic genes [20], in this study, the mRNA levels of CPT1a and ACO1 (Figure 4) and CPT enzyme activity (Figure 5) were not altered by the NP diet. Since previous studies have suggested that *db*/*db* mice are not suitable for evaluating the lipolysis-enhancing effects of functional dietary components [12], whether the EPA from NP can affect lipolysis needs to be re-evaluated in other animal models. In addition, ω3 PUFAs reportedly not only inhibited lipid accumulation in cells (which is the “first hit” of the “two-hit” hypothesis of NAFLD development) but also the inflammatory stress injury to hepatocytes, which is the “second hit” [12,20]. Therefore, we examined how NP affected the expression of inflammatory factors in the livers of *db*/*db* mice and found that the mRNA levels of MCP-1 and TNFα were significantly lower in the Np group than in the Co group (Figure 6A). The levels of liver damage markers in the serum were also significantly lower in the Np group than in the Co group (Figure 6B). These results suggest that ingestion of NP prepared by the mechanical processing of Nori effectively inhibits the progression of NAFLD.

The Fp group, which consumed the Koji-fermented FNP diet for 4 weeks, showed marked improvement in liver enlargement, hepatic steatosis, and hepatic injury in *db*/*db* mice as compared with the Co group (Table 1, Figure 3, Figure 4, Figure 5 and Figure 6). The mechanism of alleviative actions of the FNP diet was similar to that of the NP diet, but the EPA (the main bioactive component) content in FNP was only one-third that in NP (Figure 1B). A single regression analysis of the contribution of hepatic EPA incorporation to the ameliorative effect on hepatic injury also suggested that there were additional factors to EPA incorporation, by which the FNP diet effectively attenuated hepatic injury in *db*/*db* mice (Figure 8). In other words, we hypothesized that functional molecules that improve NAFLD may be produced during Koji fermentation of Nori. In fact, Koji fermentation reportedly produces a variety of bioactive substances in grains [21,22,23,24].

Metabolomic analysis of NP and FNP using CE-TOF MS and PCA of metabolite changes revealed that three betaine analogs (stachydrine, betaine, and carnitine) were predominantly produced by Koji fermentation of Nori (Figure 8). Betaine is a general term for compounds with a positive charge and a negative charge in non-adjacent positions within the same molecule, with no dissociable hydrogen bonded to the positively charged atoms and no charge on the molecule as a whole. Stachydrine, known as proline betaine and (2S)-1,1-dimethylpyrrolidinium-2-carboxylate, one of the main components of the medicinal plant *Leonurus heterophyllus*, has demonstrated various bioactivities for treating several diseases, including NAFLD [25]. Stachydrine, in particular, exhibits anti-inflammatory properties by inhibiting the nuclear factor-κB signaling pathway and is expected to be used therapeutically against cardiovascular diseases [25,26,27]. Betaine, known as glycine betaine and trimethylammonioacetate, was named after its discovery in sugar beet (*Beta vulgaris subsp. vulgaris*) and has also been reported to have preventive and therapeutic effects on liver diseases [28,29,30]. The improvement in liver diseases by betaine is attributed to a variety of molecular mechanisms, including inhibiting the inflammatory response and fatty acid synthesis [29,30]. Carnitine, also known as lysine betaine and γ-trimethyl-β-oxybutyrobetain, is synthesized from lysine and methionine mainly in the liver of animals, including humans, and may have a therapeutic role in liver diseases, like NAFLD [31,32]. Many studies have suggested that L-carnitine treatment reduces the fat accumulation in the liver by increasing fatty acid β-oxidation [31,32]. As these betaines are present in a considerable amount in FNP, we believe that their bioactivity may contribute to the therapeutic effect of the FNP diet on hepatic steatosis and liver injury in *db*/*db* mice.

The main limitation of this study is that we fed the mice with high doses of both NP and FNP and focused on the uptake of EPA into the body over a short feeding period. Further studies wherein model animals are fed a low-volume supplemented diet for a longer feeding period could provide more insights into the robustness of the test and its practicality for the human diet. Additional studies are necessary to analyze the inhibitory effect of the ingestion of both the powders on NAFLD progression, in conjunction with more detailed histological evaluation (Oil Red O, CD45, or F4/80 staining, etc.), using animal models of NAFLD that develop steatohepatitis and advancing fibrosis [8].

In conclusion, we suggested that (1) mechanical processing of Nori makes its lipid components readily absorbable by the body to exert their lipid-lowering effects, and (2) fermentation of Nori produces anti-inflammatory and lipid-lowering molecules, which, together with functional lipid components, also exert their hepatic steatosis-alleviating effects.

## 4. Materials and Methods

### 4.1. Preparation and Characterization of Nori Powder and Fermented Nori Powder

Nori sheets, a hot-airdried form of the seaweed Neopyropia yezoensis, were supplied by JA Saga (Saga, Japan). NP was prepared using a hammer crusher (Sansho Industry Co., Ltd., Osaka, Japan) and FNP was prepared by Koji fermentation of Nori using Aspergillus oryzae (Maruhide Shoyu Co., Ltd., Saga, Japan) [33]. The sample powders were characterized using SEM and gas chromatography-mass spectrometry (GC-MS). Lipids from the powders were extracted as described by Bligh and Dyer [34]. The powders were coated with OsO4 using an osmium coater (HPC-1S, Vacuum Devices Co. Ltd., Mito, Ibaraki, Japan), and images were obtained using a JSM-7500F scanning electron microscope (JEOL Ltd., Tokyo, Japan). GC-MS conditions were as follows: equipment, GC2010 + QP2010 (Shimadzu Corp., Tokyo, Japan); column, SP2380 (L 100 m × I.D. 0.25 mm × d 0.2 μm, Sigma-Aldrich Co., Tokyo, Japan); carrier gas flow rate, 20 cm/s (helium); column temperature, 140 °C for 5 min, 140–205 °C at 1.5 °C/ min, 205–240 °C at 10 °C/min, 240 °C for 8 min; injection, 1 μL; split ratio 20:1 at 250 °C; ion source, electron ionization. Samples and standards were treated with 0.5 M potassium hydroxide-methanol solution (100 °C for 9 min) followed by 10% BF_3_-methanol solution (100 °C for 7 min) to derivatize into fatty acid methyl esters prior to GC-MS.

### 4.2. Animals

All experiments were conducted under with the guidelines provided by the Ethical Committee for Experimental Animal Care at Saga University (Certificate Number: 28-058-1). Six-week-old male *db*/*db* mice were purchased from Japan SLC (Shizuoka, Japan). The mice were housed individually in plastic cages in a temperature-controlled room (24°C) under a 12-h light/dark cycle. Basal semisynthetic diets were prepared according to the recommendations of the American Institute of Nutrition (AIN-76 ^TM^) [35] and they contained (in weight %): casein, 20; cornstarch, 15; cellulose, 5; vitamin mixture (AIN-76^TM^), 1; mineral mixture (AIN-76^TM^), 3.5; DL-methionine, 0.3; choline bitartrate, 0.2; corn oil, 7; and sucrose, 48. The *db*/*db* mice were divided into three groups that were fed one of three diets: a basal semisynthetic AIN-76 diet (Co group, *n* = 5), a semisynthetic AIN-76 diet supplemented with 10% Nori powder at the expense of sucrose (Np group, *n* = 6), or a semisynthetic AIN-76 diet supplemented with 10% fermented Nori powder at the expense of sucrose (Fp group, *n* = 6). The mice were fed the diets ad libitum using Rodent CAFE (KBT Oriental, Saga, Japan) for four weeks. At the end of the feeding period, the mice were sacrificed by exsanguination from the heart under isoflurane anesthesia after a 9-h starvation period. WAT and liver were excised immediately, and the serum was separated from the blood.

### 4.3. Physiological and Histochemical Evaluation of Serum and Livers

Lipids from serum was extracted by the method of Bligh and Dyer [34] and from liver was extracted using the method described by Folch et al. [36]. Measurement of fatty acid contents in the serum and liver were carried out using GC-MS. Hematoxylin and eosin staining was carried out to microscopically evaluate the degree of hepatic steatosis and hepatic triglyceride concentrations were quantified using the method of Fletcher et al. [37] Alanine aminotransferase (ALT) activity in the serum was analyzed using a Transaminase CII-test Wako kit (Wako Pure Chemical Industries, Ltd., Osaka, Japan).

### 4.4. Assays of Hepatic Enzyme Activity

Liver cytosol and mitochondrial fractions were prepared by homogenization and centrifugation. The protein concentration of each fraction was analyzed by the method of Lowry [38]. The enzyme activity of FAS [39] and G6PDH [40] in cytosol fraction were measured by the method of Kelly. Malic enzyme in cytosol fraction and CPT in mitochondrial fraction were assayed by the method of Ochoa [41] and Markwell et al. [42], respectively.

### 4.5. Analysis of mRNA Expression in the Liver

Total RNA was extracted from 100 mg of liver using the RNeasy Lipid Tissue Mini Kit (Qiagen, Tokyo, Japan). TaqMan Universal PCR Master Mix (Applied Biosystems, Tokyo, Japan) and Assay-on-Demand, Gene Expression Products (Mn01304289_m1 for ACC1, Mn00662319_m1 for FAS, Mn00550438_m1 for CPT1a, Mn00443579_m1 for ACO1, Mn00441242_m1 for MCP1, Mn00443258_m1 for TNFα, and Mm04277571_s1 for 18S rRNA, all from Applied Biosystems, Tokyo, Japan) were used for quantitative RT-PCR analysis of *ACC1*, *FAS*, *CPT1a*, *ACO1*, *MCP1*, *TNFα*, and *18S RNA* expression in the liver. Amplifications were carried out using an ABI Prism 7000 RT-PCR sequence detection system (Applied Biosystems, Tokyo, Japan).

### 4.6. Capillary Electrophoresis Mass Spectrometry

NP and FNP (10mg each) were suspended in five hundred microliters of methanol containing 8 µM of internal standards (methionine sulfone for cation and camphor 10-sulfonic acid for anion analyses), 500 µL of chloroform and 200 µL of Milli-Q water, and centrifuged (20,400× *g*, 3 min, 4 °C). The upper layer of each solution was transferred to a 1.5-mL test tube, evaporated for 30 min with a centrifugal concentrator and then separated into two layers. The upper layer was centrifugally filtered through a PALL Nanosep 3-kD cutoff filter at 9100× *g* at 4 °C. The filtrate was dried with the centrifugal concentrator. The residue was dissolved in 20 µL of Milli-Q water containing 200 μM of internal standards (3-aminopyrrolidine for cation and trimesic acid for anion analyses). The CE-MS system and its conditions were as described by Oikawa et al. [43]. All CE-TOFMS experiments were performed using an Agilent G7100A CE Instrument (Agilent Technologies, Sacramento, CA, USA), an Agilent G6224A TOF LC/MS system, an Agilent 1200 Infinity series G1311C Quad Pump VL, and the G1603A Agilent CE-MS adapter and G1607A Agilent CE-ESI-MS sprayer kit. The G1601BA 3D-CE ChemStation software for CE and G3335-64002 MH Workstation were used. Separations were carried out using a fused silica capillary (50 μm i.d. × 100 cm total length) filled with 1 M formic acid for cation analyses or with 20 mM ammonium formate (pH 10.0) for anion analyses as the electrolyte. The sample solutions were injected at 50 mbar for 15 s (15 nL). Prior to each run, the capillary was flushed with the electrolyte for 5 min. The applied voltage was set at 30 kV. The capillary temperature was maintained at 20 °C, and the sample tray was cooled to below 4 °C. Fifty percent (*v*/*v*) methanol/water containing 0.5 μM reserpine was delivered as the sheath liquid at 10 μL/min. ESI-TOF MS was conducted in the positive ion mode for cation analyses or in the negative ion mode for anion analyses, and the capillary voltage was set at 4 kV. The flow rate of the heated dry nitrogen gas (heater temperature, 300 °C) was maintained at 10 psig. In TOF MS, the fragmentor, skimmer and Oct RFV voltages were set at 110 V, 50 V and 160 V for cation analyses or at 120 V, 60 V and 220 V for anion analyses, respectively. Each acquired spectrum was automatically recalibrated using the reference masses of reference standards. The methanol dimer ion ([2M + H]^+^, *m*/*z* = 65.0597) and reserpine ([M + H] ^+^, *m*/*z* = 609.2806) for cation analyses or the formic acid dimer ion ([2M − H]^−^, *m*/*z* = 91.0037) and reserpine ([M − H]^−^, *m*/*z* = 607.2661) for anion analyses provided the lock mass for exact mass measurements. Exact mass data were acquired at a rate of 1.5 cycles/s in the range of 50–1000 *m*/*z*. Metabolites were identified and quantified as described by Oikawa et al. [43]

### 4.7. Statistical Analysis

All data values were expressed as the mean ± standard error. To assess differences among the three groups, data were analyzed by one-way ANOVA, and all differences were analyzed by the Fisher’s LSD post hoc test using the KaleidaGraph software version 4.5 (Synergy Software, Reading, PA, USA). Student’s *t*-test was used to determine the statistical significance of the differences between the data from Nori powder and fermented Nori powder. Pearson’s correlation coefficient was calculated and linear regression analysis was also carried out using the StatPlus:mac Pro v. 8.0.1.0 (AnalystSoft Inc., Walnut, CA, USA). Differences were considered significant at *p* < 0.05. Principal component analysis (PCA) and heatmap clustering of altered metabolic profiling analysis were performed using MetaboAnalyst 5.0 (https://www.metaboanalyst.ca/, last accessed on 28 March 2022).

## Figures and Tables

**Table 1:** Effect of experimental diet on growth parameters in *db*/*db* mice.

	Co	Np	Fp
Initial body weight (g)	34.8 ± 0.4	34.9 ± 0.4	34.9 ± 0.3
Final body weight (g)	42.0 ± 1.0	40.6 ± 1.4	40.4 ± 1.1
Body weight gain (g)	7.16 ± 0.64	5.77 ± 1.13	5.55 ± 0.81
Food intake (g)	142 ± 3	141 ± 1	141 ± 4
Liver weight (g/100 g body weight)	6.87 ± 0.32 ^a^	4.87 ± 0.13 ^b^	5.15 ± 0.37 ^b^
White adipose tissue weight (g/100 g body weight)			
Total	8.00 ± 0.36	8.20 ± 0.19	8.06 ± 0.34
Epididymal	4.60 ± 0.10	4.61 ± 0.13	4.87 ± 0.25
Perirenal	3.39 ± 0.29	3.59 ± 0.18	3.19 ± 0.12

^ab^ Different superscript letters show significant differences at *p* < 0.05. Co group: mice fed a basal diet (*n* = 5), Np group: mice fed a 10% NP-supplemented diet (*n* = 6), Fp group: mice fed a 10% FNP-supplemented diet (*n* = 6).

## Data Availability

The datasets used and/or analyzed during the current study are available from the corresponding author.

## Supplementary Materials

The following supporting information can be downloaded at: https://www.mdpi.com/article/10.3390/molecules27092640/s1, Table S1: Fatty acid contents of Nori powder and Fermented Nori powder; Table S2: Fatty acid contents of serum lipids in *db*/*db* mice; Table S3: Fatty acid contents of hepatic lipids in *db*/*db* mice.

## Sample Availability

Samples of the compounds are not available from the authors.
