# Supplementary material for: Dried and Fermented Powders of Edible Algae (Neopyropia yezoensis) Attenuate Hepatic Steatosis in Obese Mice
Source: Molecules. 2022 Apr 20;27(9):2640. doi: 10.3390/molecules27092640 (PMC9099931; doi:10.3390/molecules27092640)
Supplement: Supplementary file 1 [file molecules-27-02640-s001.zip › molecules-1683576-supplementary.pdf]

Supplementary Table S1.Fatty acid contents of Nori powder and Fermented Nori powder.

	Nori powder	Fermented Nori powder
	(mg/g powder)	
C10:0	0.345±0.068	0.622±0.289
C12:0	0.0362±0.0054	0.0279±0.0042
C14:0	0.0947±0.0025	0.0668±0.0037 *
C15:0	0.0342±0.0023	0.0380±0.0042
C16:0	10.2±0.2	4.81±0.12 *
C17:0	0.0239±0.0055	0.0382±0.0042
C18:0	0.374±0.010	0.321±0.018
C18:1n9c	1.19±0.04	1.42±0.02 *
C18:2n6	0.696±0.045	2.23±0.04 *
C20:1n9	1.16±0.02	0.601±0.042 *
C20:2	0.405±0.021	n.d.
C20:3n6	0.634±0.019	0.237±0.038 *
C20:4n6	0.756±0.053	0.262±0.037 *
C20:5n3	19.7±0.3	5.87±0.6 *

Each value represents mean ± SE (n = 3).
Asterisk shows significant difference at $p < 0.05$.

Supplementary Table S2. Fatty acid contents of serum lipids in *db/db* mice.

	Co	Np	Fp
		(mg/dL)	
C10:0	0.0845±0.0250	0.107±0.040	0.122±0.013
C11:0	0.0232±0.0111	0.0430±0.0248	0.0532±0.0109
C12:0	0.267±0.063	0.362±0.111	0.296±0.030
C13:0	0.0401±0.0130	0.0485±0.0113	0.0270±0.0026
C14:0	1.86±0.24	1.81±0.17	1.35±0.09
C15:0	0.481±0.118 ^{ab}	0.606±0.087 ^{a}	0.309±0.028 ^{b}
C16:0	88.6±5.0	79.8±3.7	78.2±4.1
C16:1	10.1±0.7	8.30±0.59	8.88±0.55
C17:0	0.491±0.034	0.649±0.059	0.604±0.050
C18:0	59.8±3.2 ^{a}	45.7±2.6 ^{b}	43.8±2.8 ^{b}
C18:1n9t	0.160±0.015	0.128±0.028	0.126±0.024
C18:1n9c	74.9±3.2 ^{a}	50.8±3.2 ^{b}	66.6±5.6 ^{a}
C18:2n6	109±5	106±5	110±5
C18:3n6	1.58±0.12 ^{a}	1.08±0.04 ^{b}	1.16±0.07 ^{b}
C18:3n3	0.533±0.077 ^{a}	0.948±0.155 ^{ab}	0.969±0.091 ^{b}
C20:0	0.363±0.063	0.309±0.029	0.238±0.025
C20:1n9	1.09±0.05 ^{a}	0.635±0.045 ^{b}	0.770±0.096 ^{b}
C20:2	0.517±0.030	0.508±0.020	0.552±0.015
C20:3n6	13.5±1.1 ^{a}	6.27±0.80 ^{b}	7.60±0.66 ^{b}
C20:4n6	101±10 ^{a}	46.2±3.5 ^{b}	53.5±3.4 ^{b}
C20:5n3	1.48±0.12 ^{a}	24.3±1.6 ^{b}	8.78±0.92 ^{c}
C22:0	0.448±0.311	0.118±0.007	0.0709±0.0062
C22:6n3	22.5±2.2	19.2±1.7	18.2±1.1
C24:0	1.09±0.97	0.158±0.013	0.0512±0.0078

Each value represents mean ± SE (n = 5–6). Different superscript letters show significant difference at $p < 0.05$.

Supplementary Table S3. Fatty acid contents of hepatic lipids in *db/db* mice.

	Co	Np	Fp
		(mg/g liver)	
C10:0	0.0606±0.0148	0.0445±0.0060	0.0415±0.0024
C11:0	0.0193±0.0052	0.0119±0.0023	0.0107±0.0013
C12:0	0.141±0.017 ^{a}	0.0977±0.0075 ^{b}	0.0904±0.0036 ^{b}
C13:0	0.0240±0.0083	0.0165±0.0033	0.0169±0.0027
C14:0	1.81±0.14 ^{a}	0.824±0.097 ^{b}	0.798±0.088 ^{b}
C15:0	0.0991±0.0077 ^{a}	0.0743±0.0028 ^{b}	0.0792±0.0075 ^{ab}
C16:0	77.7±6.3 ^{a}	35.9±3.9 ^{b}	37.4±4.2 ^{b}
C17:0	0.131±0.007 ^{a}	0.106±0.005 ^{b}	0.114±0.003 ^{ab}
C16:1	13.8±1.0 ^{a}	5.64±0.86 ^{b}	4.88±0.66 ^{b}
C18:0	8.92±0.59 ^{a}	5.95±0.20 ^{b}	6.72±0.29 ^{b}
C18:1n9	154±11 ^{a}	60.8±9.4 ^{b}	76.2±12.3 ^{b}
C18:2n6	36.5±2.6 ^{a}	23.4±0.7 ^{b}	23.0±0.9 ^{b}
C18:3n6	0.541±0.041 ^{a}	0.247±0.007 ^{b}	0.251±0.013 ^{b}
C18:3n3	0.330±0.034	0.347±0.023	0.275±0.014
C20:0	0.185±0.038 ^{a}	0.0986±0.0183 ^{b}	0.100±0.014 ^{b}
C20:1n9	2.74±0.44 ^{a}	0.886±0.169 ^{b}	1.48±0.31 ^{b}
C20:2	0.270±0.028	0.216±0.009	0.216±0.016
C20:3n6	0.859±0.061 ^{a}	0.599±0.043 ^{b}	0.789±0.039 ^{a}
C20:4n6	3.08±0.17 ^{a}	2.52±0.13 ^{b}	3.13±0.11 ^{a}
C20:5n3	0.0515±0.0076 ^{a}	0.681±0.073 ^{b}	0.276±0.046 ^{c}
C22:6n3	1.27±0.09 ^{a}	1.68±0.13 ^{b}	1.76±0.11 ^{b}

Each value represents mean ± SE (n = 5–6). Different superscript letters show significant difference at $p < 0.05$.
